# The validation of the visual analogue scale for patient satisfaction after total hip arthroplasty

**DOI:** 10.1007/s12570-012-0100-3

**Published:** 2012-04-12

**Authors:** Roy B. G. Brokelman, Daniel Haverkamp, Corné van Loon, Annemiek Hol, Albert van Kampen, Rene Veth

**Affiliations:** 1 Department of Orthopaedic Surgery, Rijnstate Hospital, PO Box 9555, 6800 TA Arnhem, the Netherlands; 2 Department of Orthopaedic Surgery, Academic Medical Centre, Amsterdam, the Netherlands; 3 Department of Orthopaedic Surgery, University Medical Centre, Nijmegen, the Netherlands

**Keywords:** Satisfaction, Visual analogue scale, Validation, Arthroplasty, Hip

## Abstract

**Introduction:**

Patient satisfaction becomes more important in our modern health care system. The assessment of satisfaction is difficult because it is a multifactorial item for which no golden standard exists. One of the potential methods of measuring satisfaction is by using the well-known visual analogue scale (VAS). In this study, we validated VAS for satisfaction.

**Patient and methods:**

In this prospective study, we studied 147 patients (153 hips). The construct validity was measured using the Spearman correlation test that compares the satisfaction VAS with the Harris hip score, pain VAS at rest and during activity, Oxford hip score, Short Form 36 and Western Ontario McMaster Universities Osteoarthritis Index. The reliability was tested using the intra-class coefficient.

**Results:**

The Pearson correlation test showed correlations in the range of 0.40–0.80. The satisfaction VAS had a high correlation between the pain VAS and Oxford hip score, which could mean that pain is one of the most important factors in patient satisfaction. The intra-class coefficient was 0.95.

**Conclusions:**

There is a moderate to mark degree of correlation between the satisfaction VAS and the currently available subjective and objective scoring systems. The intra-class coefficient of 0.95 indicates an excellent test–retest reliability. The VAS satisfaction is a simple instrument to quantify the satisfaction of a patient after total hip arthroplasty. In this study, we showed that the satisfaction VAS has a good validity and reliability.

## Introduction

The World Health Organisation has directly tied health with patient satisfaction: “Health is not only the absence of infirmity and disease but also a state of physical, mental and social well-being.” Greater patient satisfaction reflects better quality of care, leading to better quality of life. The visual analogue scale (VAS) is a simple and frequently used method to evaluate variations in pain intensity [6]. The assessment of pain is difficult because it is a multifaceted expression of affective, cognitive, physical, sensory, behavioural, social–cultural and subjective feelings. Despite this diversity, the VAS pain is widely used in the literature and clinical practice. The same multifaceted difficulty is present in the rating of patient satisfaction after an arthroplasty. It is essential for providers of health care services, in particular doctors, to be able to demonstrate the efficacy of treatment [15, 17]. In orthopaedic surgery, the outcome of total joint arthroplasties is traditionally evaluated by objective clinical methods that are based on the assessment of pain and functional disability, scored by the orthopaedic surgeon [5, 9]. In our modern health care system, it is important to include the patient’s opinion in the quality outcome assessment of joint arthroplasty, since the patient is the most prominent participant [2, 16, 22]. In literature, there are no validated scoring systems to evaluate the satisfaction of the patients after an arthroplasty. A scoring system has to be simple to use and validated to be practical in a clinical setting. Haverkamp et al. showed in 2008 that a single question of Likert scale satisfaction questions lacks validity for hip arthroplasty patients [13]. The discussion whether in some questions a VAS or a Likert scale should be used was tackled in 1987 by Guyatt et al., showing a greater improvement in the VAS and a greater variability in the improvement on VAS compared to the Likert scale [11]. For satisfaction measurement, this could mean that a VAS system is perhaps more suitable. The satisfaction VAS has been used in recent literature, but the validation and reliability are unknown [4]. The goals of the present study were to assess the validity and reliability of the patients’ satisfaction VAS score after total hip arthroplasty.

## Patients and methods

Between October 2003 and June 2005, 189 primary total hip arthroplasties (THAs) were carried out in 180 patients (122 women and 58 men) in our joint care project. At this time, the preoperative pain VAS at rest and during activity, the Western Ontario McMaster Universities osteoarthritis index (WOMAC) and Harris hip score (HHS) were obtained. The patients gave their informed consent for this prospective study. The average age at time of operation was 67.5 years (SD 9.2). Most hips were affected by primary OA (161), 15 hips by rheumatoid arthritis and 13 hips by osteonecrosis of the femoral head. Seventy patients (75 hips) received the Charnley Elite Plus total hip prosthesis (DePuy/Johnson & Johnson, Leeds, UK). One hundred and ten patients (114 hips) received the Zweymüller hip prosthesis (Zimmer, Winterthur, Switzerland). At an average follow-up of 2.5 years (SD 0.2), 4 patients (5 hips) had died, 4 patients (4 hips) were revised and 25 patients (27 hips) were excluded because they were not able to fill in the evaluation forms because of cognitive deficit or they were not able to come to the outpatient department. The latter 25 patients (27 hips) had no complaints of their THA and had had no revision surgery. One hundred and fifty-three hips in 147 patients were clinically and radiographically assessed in the outpatient department. The patients were contacted by phone, and the goal of our study was explained. Thereafter, we send them, 4 weeks before the appointment, an envelope containing the evaluation forms. The evaluation forms consist of the WOMAC form [3, 19], pain VAS at rest and during activity [7, 18], satisfaction VAS, the Short Form 36 (SF-36) [1, 14], HHS [12] and the Oxford hip score (OHS) [10]. At arrival at the outpatient department, we received the forms, and the patients were sent to the radiology department for an AP radiograph of the pelvis. In the waiting room of this department, the patients were asked to fill in another satisfaction VAS and a VAS pain at rest and during activity. An experienced and independent physical therapist (AH) took the forms at the outpatient department and verified if they were filled in correctly. Thereafter, she performed the history and clinical examination and completed the HHS [12].

### Satisfaction visual analogue scale

A VAS for satisfaction is a horizontal line of 100-mm long. At the beginning and at the end, there are two descriptors representing extremes of satisfaction (i.e. no satisfaction and extreme satisfaction). The patient rated his satisfaction by making a vertical mark on the 100-mm line. The measurement in millimetres was converted to the same number of points ranging from 0 to 100 points. The exact question was “Are you satisfied with your hip prosthesis?” A standard explanation of how to fill in the VAS form was mentioned beneath the VAS horizontal line. The VAS form is shown in the annex.

### Construct validity

In the absence of a golden standard, the construct validity should be considered with scores that correlate well with the satisfaction VAS to judge its validity. Construct validity of the satisfaction VAS is established by comparison with the relevant components of the SF-36, VAS pain during rest and activity, WOMAC, OHS and HHS by means of Spearman’s correlation analysis. Convergent and discriminant validities are two aspects of construct validity. Convergent validity refers to the extent to which different ways of measuring the same trait intercorrelate with one another. Discriminant validity involves demonstrating that a measure does not correlate too strongly with measures that are intended to indicate different traits than it does. To measure convergent and divergent validity, satisfaction is compared by means of Spearman’s correlation analysis against the physical and mental scores from the SF-36.

### Content validity

One of the items of content validity is the presence of a floor or ceiling effect, meaning that patients score the lowest or highest possible score. Although in case of satisfaction a large ceiling effect may look as a perfect outcome of your surgery, it may make your score less usable since there is no possibility to discriminate between these patients based on this outcome alone and since ceiling effect of the VAS satisfaction, but also possible ceiling effects of the scores used for calculation of the construct validity can influence calculation of the construct validity. For all scores used, the ceiling effect is calculated.

### Reliability

Reliability refers to the fact that the same outcome should be reached if the test is performed again by the same patients when the symptoms have not changed [8]. Reliability is the basic requirement of all scientific requirements [21]. The test–retest reliability of the satisfaction VAS was determined by giving the patients a second satisfaction VAS at the outpatient department after they had handed over the completed forms which they had filled in at home. The patients did not know that they had to fill out VAS forms for the second time. All patients confirmed that they had filled the first forms more than 2 days before they went to the outpatient department. The test–retest reliability was investigated by assessing the intra-class coefficient. The intra-class correlation coefficient (ranging between 0 and 1) is an index of concordance for continuous data. An intra-class correlation coefficient of less than 0.4 is considered poor; between 0.4 and 0.75, fair; and greater than 0.75, excellent [15]. The systematic difference between the initial and the retest satisfaction VAS test determines the reliability of the test.

### Statistics

A priori power analyse on the outcome measure satisfaction indicated that with power set to 80 % and alpha set to 0.05, 150 patients were needed to validate the satisfaction VAS. The construct validity of the satisfaction VAS was tested using the Spearman rho correlation coefficients, which compare the patient satisfaction VAS with the pain VAS at rest and during activity, WOMAC, SF-36, HHS and the OHS and the improvement of the scores from preoperative to follow-up scores. The reliability was tested using the intra-class coefficients, which tested the test–retest of the satisfaction VAS. Significance was set at a *p* value <0.05.

## Results

### Patients

A total of 153 completed questionnaires were available from the follow-up. The patient satisfaction at the time of follow-up filled in at home was 85.6 (SD 25.1), and the patient satisfaction at the time of follow-up filled in at the outpatient department was 84.0 (SD 25.6). Not all patients showed an improvement in scores; the average improvements are shown in Table 1.

**Table 1:** Improvement after THA for the scores

	Mean	Standard deviation
VAS pain in rest	26.4	28.2
VAS pain during activity	46.8	28.4
WOMAC pain	34.4	22.1
WOMAC stiffness	29.7	28.2
WOMAC functioning	32.1	24.4
Harris hip score	31.0	18.9

### Construct validity

The Spearman rho compared to VAS pain varies from 0.62 to 0.80 which proves construct validity since pain is one of the most important factors in hip replacement. The construct validity compared to the WOMAC, Harris hip score and Oxford hip score are good with a rho varying between 0.53 and 0.70. Improvement compared to the preoperative status shows poor construct validity (Table 2). Convergent and divergent validity shows to be poor; both physical functioning and mental health of the SF-36 have a rho of 0.21 and 0.31 where only bodily pain scores a rho of 0.48.

**Table 2:** Construct validity, Spearman rho is shown for the VAS satisfaction against the different scores and their improvement

	VAS satisfaction
Different scores	
SF-36	
Physical components	
Physical functioning (PF)	0.31
Role limitation due to physical problems (RP)	0.40
Bodily pain (BP)	0.48
Social functioning (SF)	0.42
Mental components	
Mental health (MH)	0.21
Role limitation due to emotional problems (RE)	0.11
Energy and vitality (VT)	0.34
General health (GH)	0.30
VAS pain rest	0.62
VAS pain activity	0.80
WOMAC	
Pain	0.59
Function	0.53
Physical functioning	0.461
Oxford 12Q	0.67
Harris hip score	0.70
	
**Improvements**	
VAS pain rest	0.35
VAS pain activity	0.57
WOMAC	
Pain	0.40
Function	0.45
Physical functioning	0.54
Harris hip score	0.55

For all correlation coefficients, *p* < 0.01

### Content validity

**Fig. 1:**
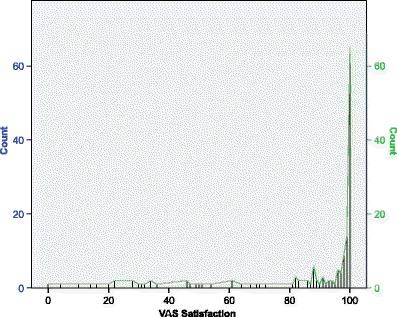
The distribution of the VAS satisfaction

A strong ceiling effect of the satisfaction VAS is present. Sixty-five patients (42 %) scored 100 on the VAS satisfaction, meaning very satisfied. The distribution of the VAS satisfaction is shown in Fig. 1. The amount of ceiling effect per score is given in Table 3.

**Table 3:** Ceiling effects per objective and subjective score

Oxford hip score	20.8 %
WOMAC pain	43.5 %
WOMAC stiffness	40.3 %
WOMAC physical	20.8 %
Harris hip score	7.1 %
VAS pain activity^a^	28.6 %
VAS pain rest^a^	33.8 %

^a^For VAS, a floor effect

### Reliability

The test–retest, measured between the patient satisfaction VAS filled in at home and the patient satisfaction VAS filled in at the outpatient department, showed an intra-class coefficient of 0.95 (95 % CI 0.93–0.96).

### Subanalysis 

Because of the high ceiling effect, we performed a subanalysis for patients scoring 95 or less on the VAS satisfaction. Construct validity and reliability remained unchanged.

## Discussion

Our goal was to assess validity and reliability of patient satisfaction administered by a visual analogue scale after total hip replacement. Our study shows good construct validity and poor content validity in the form of a high ceiling effect and good reliability for this VAS satisfaction scale in hip arthroplasty patients.

The main problem in assessing construct validity of a satisfaction score is the choice of scores to compare it with. Since the main indications for total hip arthroplasty are pain and limitations, we choose objective and subjective scoring systems (WOMAC, OHS, HHS and pain VAS at rest and during activity) to estimate construct validity. Although satisfaction is influenced by several widely differing events and complaints, for our population, pain seems to be the most suitable factor to calculate construct validity, since pain is the main indication to perform a hip arthroplasty.

Discriminant validity is also more difficult to assess since satisfaction is both influenced by physical and emotional factors. However, since we assumed in our population that physical factors would largely outweigh the emotional and psychological factors, we assessed divergent and convergent validity by comparing it to the relevant domains of the SF-36. This assumption appears to be false, since divergent and convergent validity shows to be poor using this method.

Our results show good to excellent construct validity when looking at the pain scores at follow-up with a rho varying between 0.62 and 0.80. These higher correlations were previously demonstrated between the disease-specific scores and domains of the general health questionnaires in other validation studies [9, 19, 21]. When looking at the improvement from preoperative to follow-up, these Spearman rho are much lower, indicating that satisfaction measured by a VAS scale is less suitable to postulate a statement over the amount of improvement over time but refers strongly to the current state the patient is in compared to his/her memory of the preoperative state.

One of our findings is a ceiling effect of 42 %. According to the criteria posted by Terwee et al., a ceiling effect of more than 15 % is not acceptable [20]. Previous study by Haverkamp et al. showed a ceiling effect of 79 % to be present in a Likert satisfaction scale for hip arthroplasty [13]. This indicates that for measuring satisfaction by means of a single question, the visual analogue score is clearly superior compared to a Likert scale. Although, it should be kept in mind that a ceiling effect is present for the satisfaction VAS. However, since satisfaction is not usable to measure the amount of improvement over time but gives a reflection of the patients’ well-being on that follow-up moment compared to the recollection of the preoperative status, a ceiling effect of 42 % does not render the satisfaction VAS as unusable. Furthermore, it should be noted that in our population, all scores used show a high ceiling effect, except for the Harris hip score. These high ceiling effects of all outcomes may influence calculation of construct and reliability; therefore, additional calculations were performed excluding the ceiling effects.

Reliability is usually measured by obtaining the same outcome under identical circumstances. In our study, we chose to obtain satisfaction both at home as well as on the outpatient clinic. One of the statements against using these single question satisfaction score is that the patients tend to score more satisfaction since they are more or less dependent of their surgeon for continuity of their treatment (never bite the hand that feeds you). Our test–retest reliability shows that it is not relevant where the satisfaction VAS is filled in and that obtaining it on the outpatient clinic is a reliable method.

## Conclusions

In this study, we showed that the satisfaction VAS has a good validity and reliability, however with a ceiling effect of 42 %. The VAS satisfaction is a simple and valid instrument to quantify the satisfaction of a patient after a hip arthroplasty but cannot be used as the only outcome measurement. We conclude that the VAS satisfaction is probably a useful addition to subjective and objective outcome measurements in documenting the result of total hip arthroplasty.
